# Beyond Chronic Inflammatory Demyelinating Polyradiculoneuropathy: Anti-Contactin-1 Autoimmune Nodopathy Unmasked by Proteinuria

**DOI:** 10.3390/jcm15166244

**Published:** 2026-08-12

**Authors:** Roberta Piera Bencivenga, Aniello Iovino, Maria Ucci, Emanuele Cassano, Raffaele Natella, Agnese Pecoraro, Giulia Pacella, Teresa Carandente Gianrusso, Giuseppe D’Amico, Giovanni Cerullo, Marcello Zappia, Giovanni Merola

**Affiliations:** 1 Neurology and Stroke Unit, Department of Medical Sciences, University Hospital ‘San Giovanni di Dio e Ruggi d’Aragona’, 84131 Salerno, Italy; roberta.bencivenga@live.it (R.P.B.); anielloiovino@msn.com (A.I.); agnese.pecoraro96@gmail.com (A.P.); 2 Neurology and Stroke Unit, ‘San Giuseppe Moscati’ Hospital, 81031 Aversa, Italy; ucci.maria@gmail.com (M.U.); teresa87cg@hotmail.it (T.C.G.); gius.damico90@gmail.com (G.D.); giovanni.cerullo@aslcaserta.it (G.C.); 3 Neurology and Stroke Unit, Ospedale del Mare, ASL Napoli 1 Centro, 80147 Naples, Italy; e.cassano94@gmail.com; 4 Fondazione Trotula de Ruggiero, 84121 Salerno, Italy; raffaele.natella@unimol.it; 5 Biomedical Research Centre, Gruppo Forte, 84124 Salerno, Italy; 6 Department of Medicine and Health Science “V. Tiberio”, University of Molise, 86100 Campobasso, Italy; g.pacella@studenti.unimol.it (G.P.); marcello.zappia@unimol.it (M.Z.)

**Keywords:** autoimmune nodopathy, contactin-1, anti-CNTN1 antibodies, chronic inflammatory demyelinating polyradiculoneuropathy, proteinuria, nodal/paranodal neuropathy, rituximab, B-cell-directed therapy, sensory ataxia, systemic biomarkers, case-report

## Abstract

**Background/Objectives:** Autoimmune nodopathies are a distinct subgroup of immune-mediated peripheral neuropathies caused by antibodies targeting nodal and paranodal proteins, including contactin-1 (CNTN1). These disorders are frequently misclassified as chronic inflammatory demyelinating polyradiculoneuropathy (CIDP), despite fundamental differences in pathophysiology, clinical course, and treatment response. Growing evidence indicates that systemic manifestations, such as proteinuria, may represent relevant diagnostic red flags. We report a case of anti-CNTN1 autoimmune nodopathy with renal involvement and long-term follow-up after rituximab therapy. **Methods:** We describe the longitudinal clinical, electrophysiological, laboratory, and therapeutic features of a patient presenting with an acute/subacute sensory ataxic neuropathy evolving into a chronic immune-mediated disorder. Serial nerve conduction studies were performed during multiple hospital admissions and follow-up visits. Autoantibody testing for nodal/paranodal antigens was undertaken, and systemic biomarkers were monitored over time. **Results:** The patient initially exhibited a robust response to intravenous immunoglobulin (IVIg), consistent with an acute inflammatory neuropathy. Subsequent relapse was characterized by cranial nerve involvement, worsening sensory ataxia, peripheral edema, albuminocytologic dissociation on cerebrospinal fluid analysis, proteinuria, and an inverted albumin/gamma globulin ratio, redirecting the diagnostic hypothesis toward CIDP. The detection of anti-CNTN1 antibodies ultimately established the diagnosis of autoimmune nodopathy. Owing to poor durability of IVIg, rituximab was initiated, resulting in sustained clinical improvement and near-complete recovery of motor and sensory nerve conduction parameters at one-year follow-up (November 2025). **Conclusions:** This case emphasizes the diagnostic relevance of extraneurological biomarkers, including proteinuria and peripheral edema, in autoimmune nodopathies and supports early nodal/paranodal antibody testing in atypical demyelinating neuropathies. Prompt B-cell-directed therapy may enable functional recovery of nodal integrity and improve long-term outcomes. Further studies are needed to clarify potential immunological triggers, including anti-IL-23 therapies, in the pathogenesis of anti-CNTN1 autoimmune nodopathy.

## 1. Introduction

Autoimmune nodopathies are increasingly recognized as a distinct subgroup of immune-mediated peripheral neuropathies characterized by antibodies directed against nodal and paranodal proteins, including neurofascin isoforms (NF140/155/186), contactin-1 (CNTN1), and contactin-associated protein 1 (Caspr1). These disorders differ fundamentally from classical chronic inflammatory demyelinating polyradiculoneuropathy (CIDP), as the primary immune target is the axoglial junction rather than compact myelin. This pathogenic distinction results in functional conduction block, early axonal injury, and a clinical course and therapeutic response that are distinct from those of CIDP [1,2,3].

Among autoimmune nodopathies, anti-CNTN1-associated disease is rare but clinically significant, typically presenting with prominent sensory ataxia, tremor, rapid disease progression, and early axonal involvement. Anti-CNTN1 antibodies predominantly belong to the IgG4 subclass, which does not activate complement and is generally associated with absent or transient responses to intravenous immunoglobulin (IVIg). In contrast, B-cell-directed therapies, particularly rituximab, have shown consistent and sustained efficacy, supporting a central role of B cells in disease pathogenesis [4,5,6,7].

Beyond neurological involvement, systemic manifestations have increasingly been reported in patients with nodal/paranodal antibodies. Proteinuria and membranous nephropathy have been described in association with anti-CNTN1 and anti-NF155 antibodies, suggesting a shared pathogenic mechanism involving CNTN1 expression in podocytes and disruption of the glomerular filtration barrier [8,9,10,11]. Recognition of these extraneurological biomarkers may improve diagnostic accuracy and enable earlier initiation of targeted immunotherapy.

Here, we describe a patient whose disease course evolved from an initial Guillain–Barré syndrome (GBS)-like presentation to a CIDP/DADS phenotype, ultimately diagnosed as anti-CNTN1 autoimmune nodopathy with renal involvement. We report detailed longitudinal electrophysiological and immunological follow-up, highlighting diagnostic pitfalls, therapeutic response, and the possible role of immunological triggers, including prior exposure to anti-IL-23 therapy. Collectively, these features provide a strong biological rationale for early escalation toward B-cell-directed therapeutic strategies in selected patients.

## 2. Materials and Methods

### 2.1. Clinical Assessment

Clinical data were collected prospectively during hospital admissions and outpatient follow-up. Neurological examination was performed by experienced neurologists at each time point, focusing on motor strength, sensory modalities, deep tendon reflexes, cranial nerve involvement, gait, and coordination. Disease course, relapse timing, and treatment responses were documented longitudinally.

### 2.2. Neurophysiological Studies

Nerve conduction studies (NCSs) and electromyography (EMG) were performed according to standard international guidelines using surface electrodes. Motor and sensory studies included median, ulnar, tibial, common peroneal, superficial peroneal, and sural nerves bilaterally. Evaluated parameters comprised distal motor latency, motor and sensory conduction velocities, compound muscle action potential (CMAP) amplitude, sensory nerve action potential (SNAP) amplitude, F-wave latency and persistence, and the presence of after-discharges or myokymic activity. Somatosensory evoked potentials (SSEPs) were assessed to evaluate proximal sensory pathway involvement. Serial examinations were carried out during acute presentation, relapse phases, and follow-up, including one year after rituximab therapy.

### 2.3. Laboratory and Immunological Investigations

Routine laboratory testing included complete blood count, serum chemistry, inflammatory markers, serum protein electrophoresis, and urinalysis with 24 h protein quantification. Cerebrospinal fluid analysis was performed during acute and relapse phases. Autoantibody testing for nodal and paranodal antigens (including CNTN1, NF155, and CNTN1/Caspr1 complex) was conducted using cell-based assays at the Neuroimmunology Ultra-Specialized Reference Laboratory of ‘IRCSS Fondazione Mondino, Pavia, Italy’. Peripheral blood lymphocyte subpopulations were analyzed by flow cytometry, quantifying CD3^+^ T cells, CD19^+^ B cells, CD4^+^/CD8^+^ ratio, and natural killer (NK) cells, before and after B-cell-depleting therapy.

### 2.4. Imaging

Magnetic resonance imaging (MRI) of the lumbosacral roots was performed during the initial diagnostic workup.

### 2.5. Therapeutic Interventions and Follow-Up

Therapeutic interventions included intravenous immunoglobulin (IVIg), high-dose corticosteroids, and rituximab administered as two infusions of 1000 mg separated by two weeks. Clinical, neurophysiological, and immunological follow-up was performed for up to one year after rituximab initiation.

## 3. Results

### 3.1. Case Presentation

#### 3.1.1. History

A patient with psoriatic arthritis treated with an anti-IL-23 monoclonal antibody presented in February 2024 with acute distal paresthesias, gait ataxia, areflexia and two more unusual symptoms such as lower limb myokymia and mild bilateral orbicularis oculi weakness. Neurophysiology showed myokymic discharges, absence of H-reflex and after-discharges following F-wave stimulation in all the nerves; lower-limb somatosensory evoked potentials (SSEPs) were absent. Cerebrospinal fluid (CSF) and lumbosacral magnetic resonance imaging (MRI) were unremarkable.

Given the acute presentation, a clinical diagnosis of Guillan–Barré Syndrome (GBS), bifacial weakness with paresthesia variant (BFP)/Miller–Fisher Syndrome (MFS), and acute ataxic neuropathy variant overlap [12] was initially suspected, so treatment with IVIg (0.4 g/kg/day) was started early with rapid improvement and resolution of clinical symptoms. However, anti-ganglioside antibodies were negative.

By June 2024, the patient showed a new clinical presentation with facial diplegia, dysphagia, worsening distal paresthesias, ataxia, distal weakness, especially in upper limbs, and peripheral edema. CSF revealed albuminocytologic dissociation. Neurophysiology indicated predominantly demyelinating patterns with markedly prolonged distal latencies and F-wave abnormalities, fulfilling the EAN/PNS criteria (view in Table 1) and consistent with distal acquired demyelinating symmetric (DADS) neuropathy, a CIDP variant. Laboratory tests showed proteinuria (85 mg/24 h) and inversion of the albumin/gamma globulin ratio on serum protein electrophoresis, suggesting concomitant renal and immune dysregulation. After this first relapse, treatment with the anti-IL-23 monoclonal antibody for psoriatic arthritis was discontinued.

**Figure 1:**
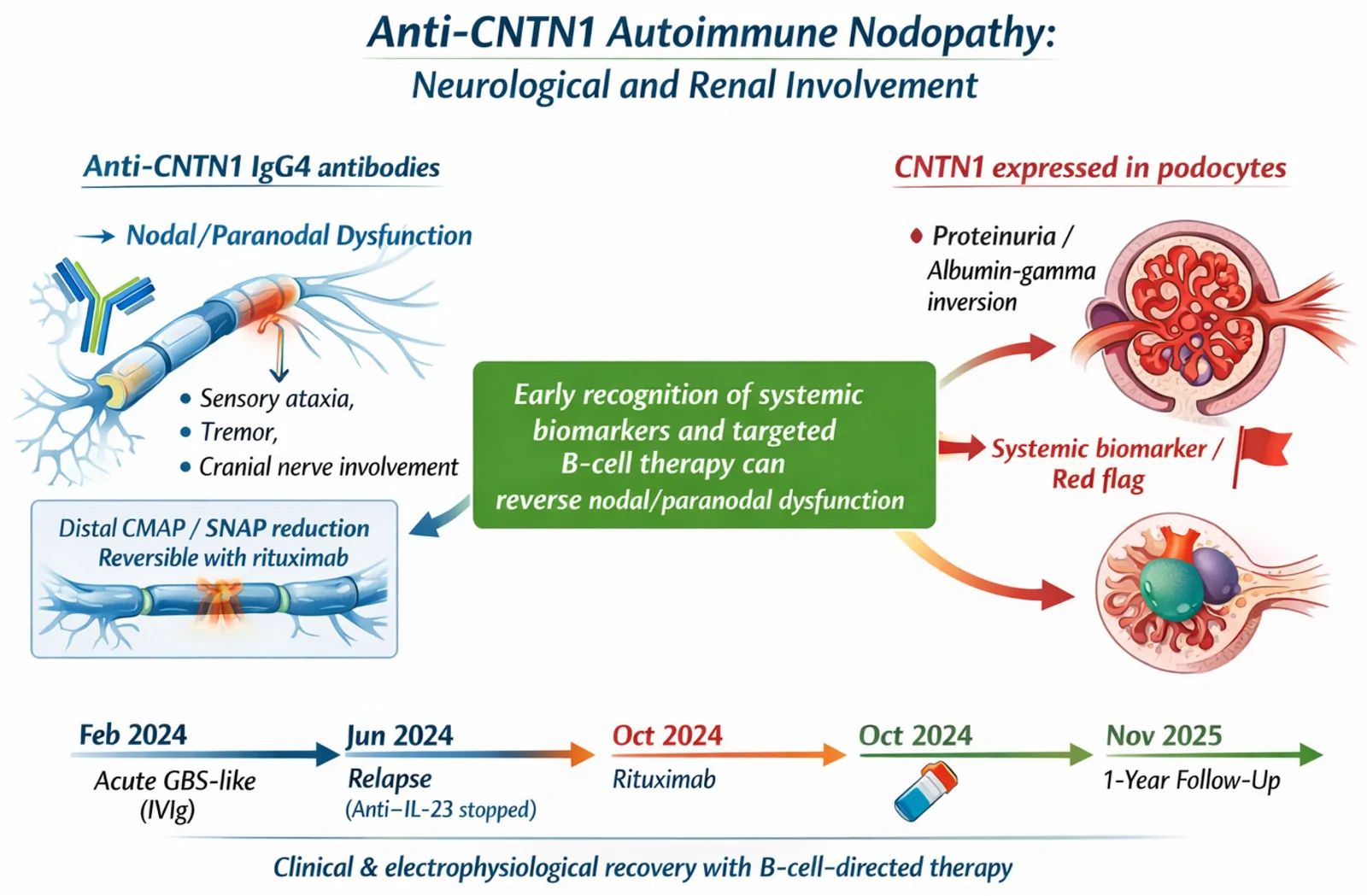
Longitudinal overview of anti-CNTN1 autoimmune nodopathy. Comprehensive visual summary of anti-CNTN1 autoimmune nodopathy, illustrating both the neurological and renal pathogenic axes. The upper left illustrates the mechanism of anti-CNTN1 IgG4-mediated axoglial junction disruption at the node of Ranvier, leading to clinical features like ataxia, tremor, and cranial nerve palsies, with associated electrophysiological reduction (CMAP/SNAP). The upper right depicts CNTN1 expression in podocytes, which serves as a crucial systemic biomarker, where proteinuria and albumin-gamma inversion are important ‘red flags’ for this disorder. The central panel highlights the importance of early diagnosis and targeted B-cell therapy in reversing dysfunction. Finally, the longitudinal timeline presents a case study from February 2024 to November 2025, charting the clinical course from an acute Guillain–Barré-like syndrome, emphasizing the pivotal role of anti-IL-23 cessation and B-cell depletion. Abbreviations: IVIg, intravenous immunoglobulin; CNTN1, Contactin-1; GBS, Guillain–Barré syndrome; CMAP, compound muscle action potential; SNAP, sensory nerve action potential.

Partial improvement followed a second course of IVIg combined with high-dose corticosteroids. Given atypical features, such as distal phenotype with dysphagia, ataxia and proteinuria, nodal/paranodal antibody testing was performed, revealing anti-CNTN1 IgG and anti-CNTN1/Caspr1 complex antibodies, confirming autoimmune nodopathy. Follow-up in July 2024 demonstrated clinical and neurophysiological stability. In October–November 2024, clinical worsening prompted initiation of rituximab (1000 mg administered twice, two weeks apart). Long-term follow-up in November 2025 demonstrated sustained clinical and electrophysiological remission. Figure 1 provides a visual longitudinal overview, summarizing key points.

#### 3.1.2. Electrophysiological Findings

Serial nerve conduction studies are summarized in Table 1, detailing motor and sensory parameters across multiple time points from first admission to long-term follow-up after rituximab. Evaluated parameters included distal motor latency (DML), motor conduction velocity (MCV), compound muscle action potential (CMAP) amplitude, F-wave latency and persistence, sensory conduction velocity (SCV), and sensory nerve action potential (SNAP) amplitude for median, ulnar, tibial, common peroneal, superficial peroneal, and sural nerves bilaterally.

Across disease evolution, electrophysiological findings demonstrated marked temporal variability. Early recordings showed prolonged distal latencies, after-discharges following F-wave stimulation, and reduced conduction velocities consistent with nodal/paranodal dysfunction. Relapse phases were associated with further distal slowing, attenuation of CMAP and SNAP amplitudes, and absent or markedly prolonged F-waves, while dispersion phenomena typical of remyelination were notably absent. Following rituximab therapy, progressive normalization of conduction velocities, recovery of CMAP and SNAP amplitudes, and restoration of F-wave parameters were observed, with near-complete electrophysiological recovery documented at one-year follow-up.

#### 3.1.3. Immunological Findings

Flow cytometric analysis of peripheral blood lymphocyte subpopulations was performed to characterize the immunological profile before and after B-cell-depleting therapy. Assessments were conducted at three time points: pre-rituximab (25 October 2024), six months post-rituximab (13 May 2025), and one year post-rituximab (30 December 2025). At six months after rituximab, complete peripheral B-cell depletion was observed (CD19^+^ B cells 0.0%; reference range 5–20%), with a relative increase in T lymphocytes (86.5%; reference range 60–80%) and preserved CD4^+^/CD8^+^ ratio (2.3). Natural killer (NK) cells accounted for 10.8% of lymphocytes, within the normal range. Longitudinal comparison across the three time points summarized in Table 2.

## 4. Discussion

The longitudinal immunological findings summarized in Table 2 reinforce the central pathogenic role of B cells in anti-CNTN1 autoimmune nodopathy. Before rituximab, peripheral B cells were detectable alongside an expanded T-cell compartment and a preserved CD4^+^/CD8^+^ ratio. Six months after treatment, complete B-cell depletion was achieved, while at one year a partial B-cell reconstitution was observed, without clinical or electrophysiological relapse. The parallel between sustained B-cell suppression, clinical stability, and electrophysiological recovery supports a mechanistic link between humoral autoimmunity and disease activity and highlights the value of serial immunophenotyping for monitoring treatment response.

### 4.1. Diagnostic Challenges and Nosological Implications

This case exemplifies the diagnostic complexity of autoimmune nodopathies, which frequently mimic Guillain–Barré syndrome or chronic inflammatory demyelinating polyradiculoneuropathy (CIDP) variants, particularly distal acquired demyelinating symmetric (DADS) neuropathy. The acute/subacute onset, normal spinal MRI, and rapid response to IVIg at first presentation were consistent with an acute inflammatory neuropathy. However, subsequent relapse, cerebrospinal fluid albuminocytologic dissociation, and progressive distal electrophysiological abnormalities gradually shifted the diagnostic perspective toward a chronic immune-mediated process, CIDP-like. However, some atypical clinical and electrophysiological data, such as rapid progression over days, cranial nerve involvement without significant proximal lower limb impairment, and the absence of remyelinating features such as temporal dispersion, have guided the search for nodal/paranodal antibodies. This evolution underscores the importance of integrating longitudinal clinical, laboratory, and neurophysiological data rather than relying on a single time-point assessment. Autoimmune nodopathies are now clearly distinguished from CIDP by their primary immune target at the nodal/paranodal axoglial junction rather than compact myelin [1,2,3]. Disruption of CNTN1–Caspr1–NF155 interactions results in functional conduction failure and secondary axonal injury, accounting for the often-rapid progression and severe disability observed in these patients and for their distinct therapeutic responsiveness [13,14].

### 4.2. Electrophysiological Correlates of Nodal/Paranodal Dysfunction

Serial nerve conduction studies provided a detailed longitudinal picture of disease evolution over more than one year. Early recordings demonstrated myokymic discharges, after-discharges following F-wave stimulation and absent or markedly delayed somatosensory evoked potentials, findings consistent with nodal dysfunction rather than classic segmental demyelination. During relapse phases, studies fulfilled electrodiagnostic criteria for a DADS-CIDP phenotype, with prolonged distal motor latencies, marked slowing, severely prolonged or absent F-waves, and progressive reduction in CMAP and SNAP amplitudes. Notably, temporal dispersion of CMAPs was consistently absent, a hallmark feature of nodo-paranodopathies, distinguishing them from typical de-remyelinating neuropathies [13,14]. This electrophysiological signature supports a primary dysfunction at the node/paranode with secondary, potentially reversible axonal impairment.

Importantly, the pattern proved dynamic and partially reversible. Transient improvements in distal latencies and conduction velocities were observed after IVIg and corticosteroid therapy, whereas relapses were marked by renewed distal slowing and amplitude reduction. Following rituximab, a gradual and sustained recovery occurred, culminating at one-year follow-up with near-normalization of conduction velocities, recovery of CMAP and SNAP amplitudes, and restoration of F-wave latencies and persistence. This evolution emphasizes that effective immunotherapy can reverse functional nodal/paranodal impairment and block axonal loss. Time is nerve, so the absence of temporal dispersion remains a key electrophysiological marker that suggests looking for nodal/paranodal antibodies.

### 4.3. Immunopathogenesis and Treatment Response

The temporal pattern of treatment response is consistent with current models of immunoglobulin subclass dynamics in anti-CNTN1 disease [15,16,17]. Early phases may involve complement-activating IgG subclasses, explaining initial IVIg responsiveness, whereas later predominance of IgG4 antibodies—characterized by functional blockade without complement activation—accounts for reduced IVIg durability and relapse. In this context, B-cell-directed therapy with rituximab directly targets the pathogenic mechanism and was associated with sustained remission and electrophysiological recovery.

The temporal association between disease activity and exposure to an anti-IL-23 monoclonal antibody for psoriatic arthritis raises the hypothesis of a permissive immunological trigger. Although causality cannot be inferred, biologic therapies are known to modulate immune homeostasis and may unmask or amplify latent autoimmunity in predisposed individuals. This observation warrants careful neurological surveillance in patients receiving cytokine-targeted therapies who develop atypical peripheral neuropathies.

### 4.4. Proteinuria as a Diagnostic Red Flag and Systemic Biomarker

A particularly relevant aspect of this case is the detection of proteinuria and inversion of the albumin/gamma globulin ratio during relapse. Proteinuria has been increasingly reported in patients with anti-CNTN1 and anti-NF155 antibodies and is now recognized as a potential systemic manifestation of nodal/paranodal autoimmunity. CNTN1 expression in podocytes suggests a shared pathogenic mechanism linking paranodal dysfunction and glomerular barrier impairment [8,9,10,17]. Even mild proteinuria should therefore be regarded as a diagnostic red flag prompting nodal/paranodal antibody testing in patients with atypical demyelinating neuropathies.

### 4.5. Clinical Implications

From a multidisciplinary perspective, this case highlights the need for heightened awareness of autoimmune nodopathies beyond the neurological domain. Extraneurological findings such as proteinuria or unexplained peripheral edema may anticipate or parallel neurological deterioration and should prompt early immunological stratification. Early recognition has prognostic relevance, as delayed initiation of targeted therapy may result in irreversible axonal damage. These observations support a shift toward precision medicine approaches in immune-mediated neuropathies, integrating systemic biomarkers with advanced immunological testing.

## 5. Conclusions

Anti-CNTN1 autoimmune nodopathy should be considered in patients with atypical or treatment-refractory demyelinating neuropathies, particularly when prominent sensory ataxia, cranial nerve involvement, or systemic features such as proteinuria and unexplained edema are present. This case illustrates how extraneurological biomarkers can serve as critical diagnostic red flags and facilitate timely nodal/paranodal antibody testing.

Long-term follow-up demonstrates that early B-cell-directed therapy with rituximab may not only stabilize disease activity but also allow substantial electrophysiological recovery, supporting the concept that nodal dysfunction can be at least partially reversible. Finally, the possible interaction between cytokine-targeted biologic therapies and nodal/paranodal autoimmunity merits further investigation through dedicated mechanistic and epidemiological studies.

## Figures and Tables

**Table 1:** Longitudinal nerve conduction study findings across admissions and follow-up evaluations.

Parameter	t.1 I Admission	t.2 I Follow-Up (March 2024)	t.3 II Admission (June 2024)	t.4 III Follow-Up (July 2024)	t.5 IV Admission (October 2024)	t.6 V Follow-Up (4 Months After RTX)	t.7 VI Follow-Up (1 y After RTX)
**Median nerve (L/R)**							
DML (ms)	3.1/3.2	4.1/-	7.7/5.8	4.7/-	8.6/-	5.6/-	4.7/-
MCV (m/s)	56.5/51.8	56.6/-	37/42	40.3/-	20.1/-	40/-	50.1/-
CMAP amplitude (mV)	10.4/6.3	7.2/-	5/4.9	6/-	3.2/-	4/-	7/-
F wave latency (ms)	After-discharges	29.7/-	NR/NR	44.2/-	NR/-	39.8/-	28.6/-
F wave persistence	NR/NR	100%/-	NR/NR	80%/-	NR/-	90%/-	90%/-
SCV (m/s)	54.9/49	49.8/-	31.7/43.3	38.9/-	NR/-	38.6/-	50/-
SNAP amplitude (uV)	15.7/15.2	11/-	5.6/3.5	2.5/-	NR/-	1.5/-	17.8/-
**Ulnar nerve (L/R)**							
DML (ms)	2.7/2.5	3.2/-	5.7/5.1	4.1/3.9	-/4.9	-/3.5	-/3.5
MCV (m/s)	56.1/65	67/-	46.2/46.1	48.5/55.3	-/25.3	-/47	-/51.4
CMAP amplitude (mV)	12.5/13.4	13	12.1/11.1	6.6/8.8	-/8.5	-/8.7	-/16.3
F wave latency (ms)	After-discharges	-/-	NR/NR	41.8/43.3	-/72.5	-/37.4	-/30.5
F wave persistence	NR/NR	-/-	NR/NR	80%/80%	-/100%	-/100%	-/100%
SCV (m/s)	51.2/46.9	46/-	32.9/31.6	-/40.4	-/19.7	-/37.3	-/43
SNAP amplitude (uV)	13.4/9.7	10.6	3.4/1.9	-/5.8	-/7.3	-/6.8	-/15
**Tibial nerve (L/R)**							
DML (ms)	3.6/3.8	-/4	9.9/10	4.5/4.9	7.3/9.7	5.3/5.3	4.8/4.2
MCV (m/s)	48.5/58	-/50.5	41/41.8	46/43.3	28.1/30.4	39.5/39.3	42.8/41.3
CMAP amplitude (mV)	9.8/10.7	-/8.8	6.5/6.2	5.3/4.5	2.3/2.6	5.5/5.9	10/8.6
F wave latency (ms)	After-discharges	-/48.2	NR/NR	62.2/65.2	NR/NR	65/64.9	57.2/55.1
F wave persistence	NR/NR	-/100%	NR/NR	100%/100%	NR/NR	100%/100%	100%/100%
**Common peroneal nerve (L/R)**							
DML (ms)	3.3/4	-/-	7.6/7.2	4.4/-	5.8/-	4.9/-	4/-
MCV (m/s)	48/49	-/-	37/43	42.6/-	317-	46.6/-	45.7/-
CMAP amplitude (mV)	9.4/6.5	-/-	1.2/10.2	5.4/-	5.3/-	6.6/-	7.1/-
**Superficial peroneal nerve (L/R)**							
SCV (m/s)	NR/NR	-/-	41.6/37.5	43/-	27.9/-	50.7/-	51/-
SNAP amplitude (uV)	NR/NR	-/-	3.9/3.7	2.5/-	3.5	2.2/-	2.7/-
**Sural nerve (L/R)**							
SCV (m/s)	46.8/51.2	-/51.9	48.8/42.4	55/-	42.1/-	41.1/-	55/-
SNAP amplitude (uV)	22/17.5	-/8.2	10.3/14	6.8	3.6/-	10.6/-	9.6/-

Values are reported for left/right nerves when available. Abbreviations: DML, distal motor latency; MCV, motor conduction velocity; CMAP, compound muscle action potential; SCV, sensory conduction velocity; SNAP, sensory nerve action potential; RTX, Rituximab.

**Table 2:** Peripheral blood lymphocyte subpopulations before and after rituximab therapy.

Time Point	CD3^+^ T Cells (%)	CD4^+^/CD8^+^ Ratio	CD19^+^ B Cells (%)	NK Cells (%)
Pre-rituximab (25 October 2024)	87.7	2.3	3.7	4.7
6 months post-rituximab (13 May 2025)	86.5	2.3	0.0	10.8
12 months post-rituximab (30 December 2025)	86.4	2.6	1.7	8.7

Abbreviations: NK, natural killer cells. Reference ranges: CD3^+^ T cells 60–80%; CD19^+^ B cells 5–20%; NK cells 4–15%; CD4^+^/CD8^+^ ratio 1.2–2.5.

## Data Availability

No new data were created.

## Abbreviations

The following abbreviations are used in this manuscript:

CIDP	Chronic Inflammatory Demyelinating Polyradiculoneuropathy
CNTN1	Contactin-1
Caspr1	Contactin-associated protein 1
GBS	Guillain–Barré Syndrome
DADS	Distal Acquired Demyelinating Symmetric neuropathy
IVIg	Intravenous Immunoglobulin
RTX	Rituximab
NCS	Nerve Conduction Studies
EMG	Electromyography
CMAP	Compound Muscle Action Potential
SNAP	Sensory Nerve Action Potential
DML	Distal Motor Latency
MCV	Motor Conduction Velocity
SCV	Sensory Conduction Velocity
CSF	Cerebrospinal Fluid
SSEP	Somatosensory Evoked Potentials
MRI	Magnetic Resonance Imaging
NK cells	Natural Killer cells
